# Trends, patterns and predictors of high-risk fertility behaviour among Indian women: evidence from National Family Health Survey

**DOI:** 10.1186/s12889-024-18046-3

**Published:** 2024-02-27

**Authors:** Pooja Singh, Kaushalendra Kumar Singh

**Affiliations:** Department of Statistics, Institute of Science, Banaras Hindu University, Varanasi, Uttar Pradesh India

**Keywords:** HRFB, India, Multinomial logistic regression, NFHS, Reproductive health

## Abstract

**Background:**

Numerous studies have demonstrated that high-risk fertility behaviour (HRFB), which includes maternal age below 18 or above 34 years, short birth intervals (less than 24 months), and high parity (birth order above 4), is associated with adverse maternal and child health outcomes. There is a substantial research gap in the domain of high-risk fertility behaviour in the Indian context. Therefore, this study is designed to investigate the current trends and patterns in the prevalence of high-risk births among Indian women, with a primary focus on identifying contributing factors associated with this prevalence.

**Methods:**

The study utilized data from the nationally representative National Family Health Survey (NFHS), which has been conducted in five rounds since 1992–93. Data from all rounds were used to assess the overall trend. However, data from the most recent round of NFHS, conducted during 2019–21, were employed to evaluate current levels and patterns of HRFB prevalence and to identify socio-economic and demographic predictors of HRFB using binomial and multinomial logistic regression models.

**Results:**

The prevalence of HRFB has exhibited a consistent decreasing pattern from 1992 to 93 to 2019–21 in India. However, 29.56% of married women continue to experience high-risk births with notably higher rates in several states (e.g., 49.85% in Meghalaya and 46.41% in Bihar). Furthermore, socio-demographic factors like wealth index, educational level, social group, religion, mass media exposure, family size, age at marriage, type and region of residence, and reproductive factors like birth intention, place and type of delivery, ANC visits and current contraceptive use were identified as significant predictors of high-risk births among women in India.

**Conclusion:**

Despite a 20.4 percentage point decline in HRFB prevalence over the past three decades, a significant proportion of women in specific regions and demographic subgroups continue to experience high-risk births. Therefore, the present study recommends interventions aimed at preventing high-risk births among women in India, with particular emphasis on states with high HRFB prevalence and women from socioeconomically disadvantaged backgrounds.

## Background

The burden of adverse pregnancy outcomes, such as death of the mother and her child, stillbirth and preterm birth, remains substantial in developing countries including India. In 2017, India accounted for 12% of global maternal deaths and approximately one-fifth of under-5 deaths worldwide [1, 2].. Additionally, maternal and child undernutrition poses a significant challenge to the public health system in India. According to the fourth round of the National Family Health Survey (NFHS-4), conducted in 2015–2016, a quarter of women of reproductive age are undernourished, and the prevalence of underweight, stunting, and wasting among children under five is 35.7, 38.4, and 21.0%, respectively [3]. Child and maternal malnutrition are reported as the primary risk factor, contributing to 68·2% of under-5 deaths and 83·0% of neonatal deaths in India [4]. The government of India has implemented various nutrition-related programs and strategies, including the Integrated Child Development Service (ICDS) And POSHAN (Prime Minister’s Overarching Scheme for Holistic Nutrition) Abhiyaan, Reproductive, Maternal, Newborn, Child and Adolescent Health (RMNCH+A) Programme, Mid-day Meal (MDM), National Food Security Mission (NFSM), among others, to address the issue of malnutrition. However, despite these efforts, India has been unable to eliminate child and maternal malnutrition.

An analysis of data from the Demographic and Health Survey (DHS) across 45 countries revealed a noteworthy positive correlation between deaths and malnutrition among children under the age of five, and specific fertility-related behavioural risk factors [5]. These behaviours are collectively referred to as high-risk fertility behaviours (HRFB) and include early or late childbearing (maternal age less than 18 years or more than 34 years), closely spaced births (birth intervals less than 24 months), and high parity (more than 4). Previous studies have also identified high-risk fertility behaviour as a significant predictor of maternal chronic undernutrition [6]. Additionally, women with chronic malnutrition are more likely to give birth to children who suffer from malnutrition, thus perpetuating a cycle of malnutrition across generations [7].

It is imperative to underscore that high-risk fertility behaviours, whether occurring independently or in combination, serve as the primary underlying causes of adverse health outcomes for both the mother and her child. For instance, childbearing at either younger or advanced ages has empirically been linked to an elevated likelihood of stillbirths, preterm births and neonatal deaths [8–12]. Furthermore, a younger maternal age stands as a recognized risk factor for child malnutrition, encompassing conditions such as low birth weight, stunting, and wasting [9, 10, 13, 14]. On the other hand, advanced maternal age is linked to higher rates of genetic abnormalities, pregnancy-related complications, and caesarean sections [15, 16].

Studies have also found an increased maternal mortality risk at both younger and older ages. In fact, a “J”-shaped relationship between age and maternal mortality has been observed, with higher mortality rates at younger ages (below 18 years) and the highest mortality rates at older ages (above 35 years) [17]. In the context of birth intervals, childbirths followed by a short interpregnancy interval are associated with increased maternal and infant mortality due to insufficient maternal recovery time. Short birth intervals can also lead to an increased risk of low birth weight and maternal anaemia. Moreover, children born from pregnancies with short intervals may confront health and developmental challenges [18–25]. Furthermore, high parity is linked to various risks, including an increased risk of maternal mortality and child undernutrition [26–29].

Several studies have been conducted to identify the levels and determinants of high-risk fertility behaviour in different developing countries, including the Democratic Republic of the Congo (DRC), Ethiopia, and Bangladesh [30–34]. These studies have identified various socio-demographic and reproductive characteristics that influence the prevalence of HRFB among women of reproductive age.

In the context of India, a previous study revealed a notable prevalence of high-risk fertility behaviours during 2015–16. The study documented that 35% of married women had at least one of the high-risk fertility behaviours, including 9.4% of women having a birth interval of less than 24 months and 8.7% of women having a birth order of more than four [35]. Another study conducted in India reported that sexual intimate partner violence is statistically associated with high-risk fertility behaviours among women in India. The same study also found that engaging in high-risk fertility behaviours is influenced by various factors, such as the mother’s level of education, rural residence, religion, prenatal care, and contraceptive use [36]. Some other studies have examined the association of HRFB with chronic undernutrition and under-five mortality in India [37, 38]. However, there is no such study that has focused primarily on HRFB among women, in terms of examining the levels, trends, and determinants of the HRFB prevalence in the country.

Understanding the determinants of HRFB is crucial for developing effective interventions to reduce its prevalence and mitigate the adverse maternal and child outcomes associated with it. Therefore, the primary motivation of this paper is to fill this knowledge gap by utilizing the data from the latest nationally representative health survey (NFHS-5). The findings of this study will enable us to render some efficient policy implications to lower the incidence of high-risk births and thereby, would help in reducing the risk of maternal and child mortality and other undesirable pregnancy outcomes.

## Methods

### Data source

The study conducted secondary data analysis using the NFHS, IR (individual record) dataset. Five rounds of NFHS have been conducted so far, since 1992–93. The latest fifth round of the survey, conducted during 2019–21, provides current estimates of basic demographic, health, and health-related indicators for each state/union territory (UT), and for 707 districts. The NFHS-5 data were collected from a nationally representative sample of residential households (HHs) selected using a stratified two-stage sampling design. Primary Sampling Units (PSUs) were selected using the 2011 census as the sampling frame. Villages in rural areas and Census Enumeration Blocks (CEBs) in urban areas served as PSUs. A total of 724,115 eligible women aged 15–49 years old from 636,699 households were interviewed with a response rate of 97% [39]. The current study is based on 176,601 ever-married women with at least one child born to them in the preceding 5 years before the survey. Respondents with missing data were excluded, resulting in a final analytic sample of 176,567 women.

### Outcome variable

Maternal HRFB was the outcome variable of this study. In accordance with the NFHS, we defined HRFB as exposure of women to any of the following four demographic risks at their last childbirth: maternal age less than 18 years or more than 34 years, the birth of order four or higher, and birth interval less than 24 months. Two forms of the outcome variable were used in the analysis, one is dichotomous in nature coded as “1” if a woman falls into one or more of the high-risk fertility behaviour categories and “0” otherwise. The other form has three categories; (1) no HRFB (if a woman did not experience any high-risk fertility behaviour), (2) single HRFB (if a woman experiences a single high-risk fertility behaviour) and (3) multiple HRFB (if a woman experiences any combination of two or more high-risk fertility behaviours during her last childbirth).

### Explanatory variables

Socio-demographic and reproductive factors that have been shown to be associated with high-risk births in previous studies were included in the study. These factors were wealth index (grouped into poorest, poorer, middle, richer and richest), educational level (categorized as no education, primary, secondary and higher education), social group (categorized as Scheduled Caste (SC), Scheduled Tribe (ST), Other Backward Class (OBC), and General Caste (rest of the population), religion (classified as Hindu, Muslim, and others (other than Hindus and Muslims), mass media exposure (classified as exposed and not exposed depending on women’s exposure to at least one of the mass media i.e. television, radio and newspapers/magazines), family size (categorized into < 5, 5 to 7 and > 7), type of residence (rural or urban), region of residence (grouped into southern, northern, eastern, western, central and northeastern regions), age at marriage (categorized into < 18 years and ≥ 18 years), last birth status (wanted or unwanted), place of delivery (health facility or home), type of delivery (caesarean or normal), ANC (Antenatal Care) visits (categorized as < 4 and 4 or more) and currently using contraceptive methods (yes or no).

### Statistical analysis

The levels, trends and patterns of HRFB prevalence among married women were illustrated through graph, maps and percentage distributions. All the percentages were weighted by sampling weights provided by DHS to correct for sample design. Chi-square test of independence was performed to assess the association between different independent variables and the HRFB prevalence. Finally, at the multivariate level, binomial and multinomial logistic regression modelling was used to identify the determining factors of HRFB among women. Prior to the regression analysis, we investigated for multicollinearity by employing Variance Inflation Factor (VIF), which indicated the absence of collinearity among the explanatory variables with a Mean VIF of 1.73 (Maximum VIF = 2.69 and Minimum VIF = 1.01).

## Results

### Background characteristics of the study sample

The background characteristics of the women who participated in the study are presented in Table 1. The proportion of women among different wealth quantiles ranges from 17.38% in the richest to 22.77% in the poorest category. Most of the women (51.48%) had education up to the secondary level, while 19.52% had no formal education. The majority of women (79.58%) were Hindu, belonged to OBC social group (43.01%) and living in households with 5–7 members (48.89%). About one-fourth of women (26.76%) had no exposure to mass media. About one-third of women (32.53%) were married before the age of 18 years and the last birth was unwanted for 7.99% of the women. 71.81% of women belonged to rural areas and the majority of women were living in the central (26.65%), eastern (25.48%) and southern (17%) regions of the country. The childbearing of about 90% of women took place in a health facility, and about one-fourth of women (23.99) had a caesarean delivery. More than two-fifth of women are observed to not be using any contraceptive methods and had less than 4 ANC visits during the last childbirth.

**Table 1:** Descriptive statistics for the sample of married women aged 15–49 years who had at least one birth in the five-year preceding the survey, India, NFHS-5 (2019–21)

Background Characteristic	Frequency	Percentage (95% CI)
**Wealth Index**		
Poorest	44,769	22.77 (22.42–23.12)
Poorer	40,420	21.04 (20.73–21.36)
Middle	34,513	19.58 (19.28–19.89)
Richer	31,012	19.23 (18.89–19.57)
Richest	25,853	17.38 (17.02–17.75)
**Education**		
No education	35,927	19.51 (19.21–19.83)
Primary	21,700	11.73 (11.51–11.96)
Secondary	92,454	51.48 (51.08–51.87)
Higher	26,486	17.27 (16.9–17.66)
**Social Group**		
General	39,135	24.47 (24.02–24.92)
Scheduled Caste	35,232	22.65 (22.22–23.08)
Scheduled Tribe	35,224	9.869 (9.61–10.13)
Other Backward Class	66,976	43.01 (42.55–43.48)
**Religion**		
Hindu	129,819	79.58 (79.05–80.09)
Muslim	25,201	15.91 (15.41–16.43)
Others	21,547	4.508 (4.34–4.68)
**Mass media exposure**		
No	48,927	26.76 (26.39–27.13)
Yes	127,640	73.24 (72.87–73.61)
**Family size**		
< 5	49,872	27.77 (27.4–28.14)
5–7	87,375	48.89 (48.51–49.28)
> 7	39,320	23.33 (23–23.67)
**Age at marriage**		
< 18	53,606	32.53 (32.18–32.89)
18 or higher	122,961	67.47 (67.11–67.82)
**Type of residence**		
Urban	37,912	28.19 (27.74–28.65)
Rural	138,655	71.81 (71.35–72.26)
**Region of Residence**		
South	22,752	17.00 (16.68–17.32)
North	33,477	13.58 (13.36–13.82)
Central	43,738	26.65 (26.33–26.97)
East	33,326	25.80 (25.44–26.16)
Northeast	27,356	4.05 (3.96–4.13)
West	15,918	12.92 (12.49–13.35)
**Last birth status**		
Wanted	163,356	92.01 (91.81–92.21)
Unwanted	13,211	7.99 (7.78–8.19)
**Place of delivery**		
Health facility	155,002	90.06 (89.82–90.3)
Home	21,565	9.94 (9.70–10.18)
**Type of delivery**		
Normal	138,842	76.01 (75.69–76.33)
Caesarean	37,725	23.99 (23.67–24.31)
**ANC visits**		
< 4	75,280	41.49 (41.06–41.93)
4 or more	101,287	58.51 (58.07–58.94)
**Currently using contraceptive methods**		
Yes	103,206	59.24 (58.85–59.63)
No	73,361	40.76 (40.37–41.15)

Numbers are unweighted, percentages are weighted

### Trends and prevalence of HRFB among Indian women

**Fig. 1:**
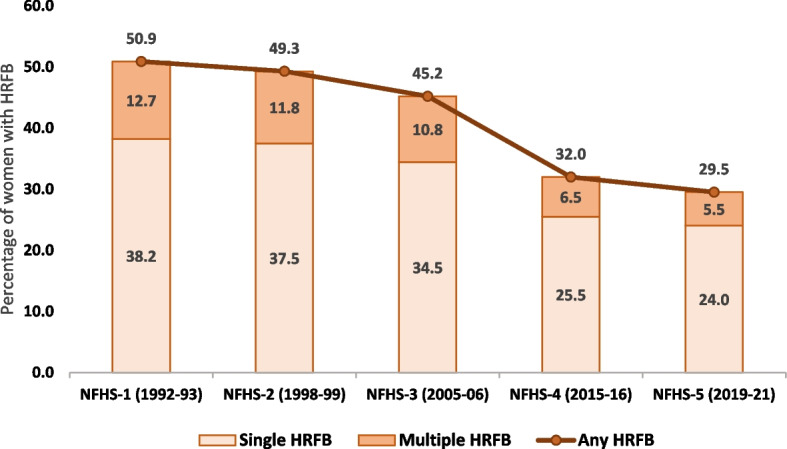
Trends in the prevalence of high-risk fertility behaviours among married women in India, NFHS-1 (1992–93) to NFHS-5 (2019–21)

Figure 1 shows the overall trends in the HRFB (single and multiple) prevalence over a period of about three decades from 1992 to 93 to 2019–21. The prevalence has shown a decreasing trend throughout the period with the percentage of women with high-risk births being declined from 50.9% during 1992–93 to 29.5 during 2019–21. Single HRFB has been reduced by 14 percentage points (from 38.2 to 24%), whereas, multiple HRFB has been reduced by 7 percentage points (from 12.7 to 5.5%). The figures from the most recent data revealed that the most common single high-risk category was birth interval less than 24 months followed by birth order four or higher. Regarding multiple HRFB, the most common combination of high-risk categories was birth interval less than 24 months and birth order more than three (Table 2).

**Table 2:** Levels of high-risk fertility behaviours among married women in India, NFHS-5 (2019–21)

High-risk fertility behaviours	Frequency	Percentage (95% CI)
Any high-risk fertility behaviour		
No	123,064	70.48 (70.14–70.81)
Yes	53,503	29.52 (29.19–29.86)
Types of high-risk fertility behaviour		
Single high-risk fertility behaviour	42,630	24.04 (23.75–24.34)
Mother’s age at birth < 18 years	2973	1.95 (1.85–2.062)
Mother’s age at birth > 34 years	3987	1.87 (1.78–1.97)
Birth interval < 24 months	21,089	12.48 (12.26–12.7)
Birth order ≥3	14,581	7.74 (7.57–7.92)
Multiple high-risk fertility behaviour	10,872	5.48 (5.33–5.63)
Age at birth < 18 years and birth interval < 24 months	263	0.18 (0.15–0.21)
Age at birth > 34 years and birth interval < 24 months	241	0.10 (0.08–0.13)
Age at birth > 34 years and birth order ≥3	4274	1.85 (1.77–1.93)
Age at birth > 34 years, birth interval < 24 months and birth order ≥3	714	0.32 (0.29–0.36)
Birth interval < 24 months and birth order ≥3	5380	3.03 (2.92–3.14)

Numbers are unweighted, percentages are weighted

### Prevalence of HRFB by states/UTs of India

**Fig. 2:**
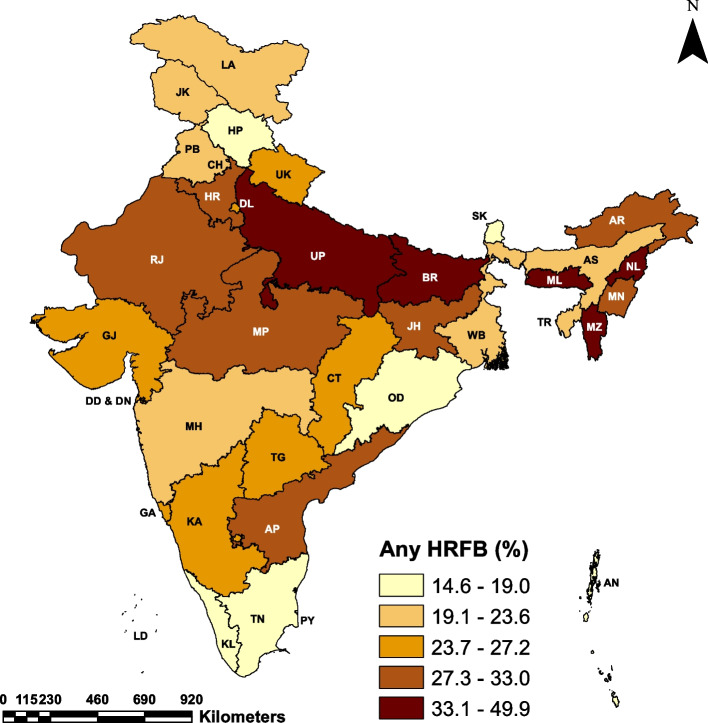
Prevalence of any HRFB among women by states/UTs of India, NFHS-5 (2019–21). Abbreviations : Andaman and Nicobar Islands = AN, Andhra Pradesh = AP, Arunachal Pradesh = AR, Assam = AS, Bihar = BR, Chandigarh = CH , Chhattisgarh = CT, Delhi = DL , Daman and Diu and Dadra & Nagar Haveli = DD & DN, Goa = GA, Gujarat = GJ, Haryana = HR, Himachal Pradesh = HP, Jammu and Kashmir = JK , Jharkhand = JH, Karnataka = KA, Kerala = KL, Ladakh = LA, Lakshadweep = LD, Madhya Pradesh = MP, Maharashtra = MH, Manipur = MN, Meghalaya = ML, Mizoram = MZ, Nagaland = NL, Odisha = OD, Puducherry = PY , Punjab = PB, Rajasthan = RJ, Sikkim = SK, Tamil Nadu = TN, Telangana = TG, Tripura = TR, Uttarakhand = U K, Uttar Pradesh = UP, West Bengal = WB

**Fig. 3:**
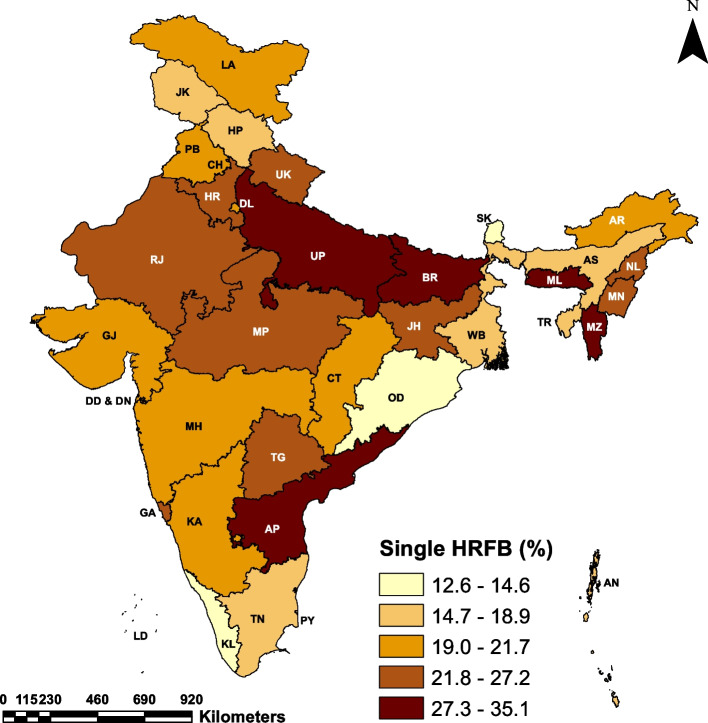
Prevalence of single HRFB among women by states/UTs of India, NFHS-5 (2019–21).  Abbreviations : Andaman and Nicobar Islands = AN, Andhra Pradesh = AP, Arunachal Pradesh = AR, Assam = AS, Bihar = BR, Chandigarh = CH , Chhattisgarh = CT, Delhi = DL , Daman and Diu and Dadra & Nagar Haveli = DD & DN, Goa = GA, Gujarat = GJ, Haryana = HR, Himachal Pradesh = HP, Jammu and Kashmir = JK , Jharkhand = JH, Karnataka = KA, Kerala = KL, Ladakh = LA, Lakshadweep = LD, Madhya Pradesh = MP, Maharashtra = MH, Manipur = MN, Meghalaya = ML, Mizoram = MZ, Nagaland = NL, Odisha = OD, Puducherry = PY , Punjab = PB, Rajasthan = RJ, Sikkim = SK, Tamil Nadu = TN, Telangana = TG, Tripura = TR, Uttarakhand = U K, Uttar Pradesh = UP, West Bengal = WB

**Fig. 4:**
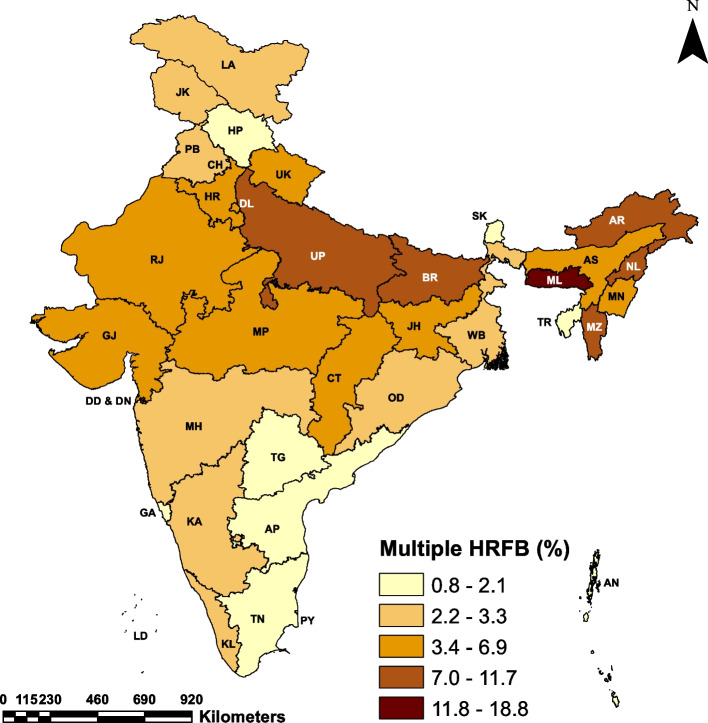
Prevalence of multiple HRFB among women by states/UTs of India, NFHS-5 (2019–21). Abbreviations : Andaman and Nicobar Islands = AN, Andhra Pradesh = AP, Arunachal Pradesh = AR, Assam = AS, Bihar = BR, Chandigarh = CH , Chhattisgarh = CT, Delhi = DL , Daman and Diu and Dadra & Nagar Haveli = DD & DN, Goa = GA, Gujarat = GJ, Haryana = HR, Himachal Pradesh = HP, Jammu and Kashmir = JK , Jharkhand = JH, Karnataka = KA, Kerala = KL, Ladakh = LA, Lakshadweep = LD, Madhya Pradesh = MP, Maharashtra = MH, Manipur = MN, Meghalaya = ML, Mizoram = MZ, Nagaland = NL, Odisha = OD, Puducherry = PY , Punjab = PB, Rajasthan = RJ, Sikkim = SK, Tamil Nadu = TN, Telangana = TG, Tripura = TR, Uttarakhand = U K, Uttar Pradesh = UP, West Bengal = WB

Table 3 presents the distribution of HRFB prevalence by the states/UTs of India. The maps in Fig. 2, Fig 3 and Fig. 4 depict the state-wise prevalence of any HRFB, single HRFB and multiple HRFB respectively. The high prevalence is represented by darker shades while lighter shades represent low prevalence of HRFB. The percentage of women who had any HRFB varies from 14.60 to 49.90% across states of India. For single and multiple HRFB, the range is 12.60 to 35.10% and 0.80 to 18.80% respectively. The state-level prevalence map also depicted that the central, eastern, and northeastern regions had a higher prevalence of HRFB among women as compared to southern and western regions. Among all the states**,** the highest percentage of women who had high-risk births comes from Meghalaya (49.9%) in the northeastern region followed by Bihar (46.4%) in the eastern region of the country. Of all the 11 states with HRFB prevalence higher than the national average (29.5%), four states are from the northeastern region (Meghalaya, Mizoram, Nagaland and Manipur), two states are from the eastern region (Bihar and Jharkhand), two states are from the central region (Uttar Pradesh and Madhya Pradesh), two states are from northern region (Haryana and Rajasthan) and only one state is from the southern region (Andhra Pradesh) of the country. The lowest prevalence of HRFB is observed in Sikkim (14.6%) followed by Odisha (16.8%). Considering multiple HRFB, five states, namely Bihar (18.8%), Andhra Pradesh (11.7%), Meghalaya (11.3%), Mizoram (10.6%) and Uttar Pradesh (9.0%) have a higher prevalence than the national average of 8.6%, whereas, Tamil Nadu showed least multiple HRFB prevalence (0.8%).

**Table 3:** Distribution of high-risk fertility behaviours by states/UTs of India, NFHS-5 (2019–21)

State/UT	Any HRFB Percentage (95% CI)	Single HRFB Percentage (95% CI)	Multiple HRFB Percentage (95% CI)
**South**			
Andaman & Nicobar Islands	17.92 (14.14–22.45)	16.4 (12.71–20.9)	1.53 (0.69–3.35)
Andhra Pradesh	31.22 (29.09–33.44)	29.53 (27.44–31.71)	1.69 (1.2–2.38)
Karnataka	24.45 (22.99–25.96)	21.72 (20.35–23.16)	2.72 (2.26–3.28)
Kerala	17.09 (15.35–18.99)	14.56 (13–16.28)	2.53 (1.86–3.43)
Lakshadweep	21.28 (17.2–26.02)	18.7 (14.74–23.43)	2.58 (1.10–5.91)
Puducherry	17.9 (13.48–23.38)	16.32 (11.97–21.84)	1.58 (0.55–4.48)
Tamil Nadu	18.88 (17.48–20.36)	18.08 (16.69–19.56)	0.79 (0.58–1.10)
Telangana	26.04 (24.36–27.8)	23.95 (22.49–25.46)	2.09 (1.54–2.85)
**North**			
Chandigarh	25.58 (17.99–35)	23.25 (16.36–31.93)	2.33 (0.89–5.96)
Delhi	25.64 (23.66–27.73)	21.71 (19.91–23.62)	3.93 (3.18–4.86)
Haryana	29.78 (28.34–31.26)	24.93 (23.64–26.27)	4.85 (4.24–5.54)
Himachal Pradesh	19.02 (16.83–21.43)	17.1 (15.1–19.3)	1.92 (1.33–2.78)
Jammu & Kashmir	22.1 (20.67–23.6)	18.77 (17.42–20.2)	3.34 (2.79–3.98)
Ladakh	23.57 (20.15–27.36)	21.13 (17.83–24.85)	2.44 (1.29–4.57)
Punjab	22.85 (21.35–24.43)	20.05 (18.63–21.55)	2.80 (2.27–3.45)
Rajasthan	29.71 (28.64–30.79)	24.08 (23.09–25.08)	5.63 (5.13–6.18)
Uttarakhand	27.22 (24.73–29.86)	22.94 (20.71–25.33)	4.28 (3.372–5.42)
**Central**			
Chhattisgarh	25.08 (23.71–26.5)	20.68 (19.45–21.96)	4.39 (3.79–5.08)
Madhya Pradesh	30.59 (29.52–31.67)	25.28 (24.33–26.25)	5.31 (4.8–5.86)
Uttar Pradesh	37.11 (36.37–37.85)	28.15 (27.5–28.8)	8.96 (8.56–9.38)
**East**			
Bihar	46.41 (45.39–47.43)	35.13 (34.2–36.06)	11.28 (10.63–11.96)
Jharkhand	32.81 (31.51–34.14)	26.49 (25.35–27.66)	6.32 (5.71–7)
Odisha	16.79 (15.74–17.9)	14.03 (13.07–15.05)	2.76 (2.37–3.22)
West Bengal	20.18 (18.6–21.86)	17.94 (16.53–19.45)	2.24 (1.8–2.79)
**Northeast**			
Arunachal Pradesh	28.84 (27.07–30.69)	20.26 (18.9–21.69)	8.59 (7.61–9.68)
Assam	22.89 (21.75–24.06)	18.56 (17.56–19.61)	4.33 (3.85–4.85)
Manipur	32.95 (30.78–35.19)	26.09 (24.17–28.11)	6.86 (5.86–8.01)
Meghalaya	49.85 (47.8–51.89)	31.09 (29.16–33.09)	18.76 (17.1–20.53)
Mizoram	40.54 (37.27–43.88)	28.86 (26.16–31.72)	11.67 (9.22–14.66)
Nagaland	37.8 (34.89–40.81)	27.18 (24.83–29.66)	10.63 (9.05–12.44)
Sikkim	14.62 (11.29–18.72)	12.61 (9.56–16.45)	2.01 (1.06–3.80)
Tripura	20.76 (18.78–22.89)	18.9 (17–20.97)	1.86 (1.32–2.60)
**West**			
Dadra & Nagar Haveli and Daman & Diu	25.22 (21.25–29.65)	20.84 (17.28–24.91)	4.38 (2.76–6.87)
Goa	26.31 (22.37–30.66)	24.23 (20.42–28.49)	2.08 (0.93–4.6)
Gujarat	24.98 (23.73–26.27)	20.39 (19.28–21.54)	4.59 (4.07–5.18)
Maharashtra	23.01 (21.26–24.85)	19.85 (18.4–21.39)	3.16 (2.59–3.86)

*UT* Union Territory, *CI* Confidence Interval.

### Prevalence of HRFB in India by background characteristics

Table 4 presents the distribution of HRFB across selected socio-economic and demographic characteristics. The results of chi-square tests revealed that all selected background variables are significantly associated (*p*-value< 0.001) with the prevalence of HRFB.

**Table 4:** Distribution of high-risk fertility behaviours by background characteristics of married women in India, NFHS-5 (2019–21)

Background Characteristic	Any HRFB Percentage (95% CI)	Single HRFB Percentage (95% CI)	Multiple HRFB Percentage (95% CI)
**Wealth Index**	*χ*^2^ = 6853.97*	*χ*^2^ = 3022.65*	*χ*^2^ = 4169.63*
Poorest	42.81 (42.17–43.46)	31.60 (31.03–32.18)	11.21 (10.81–11.63)
Poorer	33.55 (32.88–34.21)	27.11 (26.52–27.71)	6.43 (6.10–6.79)
Middle	27.67 (27.00–28.35)	23.64 (23.01–24.29)	4.03 (3.77–4.30)
Richer	22.60 (21.91–23.30)	19.97 (19.32–20.63)	2.63 (2.40–2.89)
Richest	17.00 (16.30–17.73)	15.38 (14.72–16.07)	1.62 (1.41–1.85)
**Educational level**	*χ*^2^ = 13400*	*χ*^2^ = 5178.80*	*χ*^2^ = 10200*
No education	51.57 (50.87–52.26)	35.60 (34.98–36.24)	15.96 (15.45–16.49)
Primary	37.22 (36.34–38.10)	29.64 (28.84–30.46)	7.57 (7.09–8.09)
Secondary	24.79 (24.38–25.21)	22.17 (21.78–22.56)	2.63 (2.49–2.77)
Higher	13.49 (12.89–14.12)	12.77 (12.19–13.37)	0.72 (0.59–0.88)
**Social group**	*χ*^2^ = 655.31*	*χ*^2^ = 372.49*	*χ*^2^ = 241.64*
General	25.25 (24.49–26.02)	21.04 (20.40–21.69)	4.21 (3.91–4.53)
Scheduled Caste	32.93 (32.28–33.59)	26.60 (25.99–27.22)	6.33 (6.02–6.65)
Scheduled Tribe	32.04 (31.16–32.93)	25.32 (24.54–26.11)	6.72 (6.31–7.16)
Other Backward Class	29.59 (29.12–30.06)	24.11 (23.69–24.55)	5.47 (5.26–5.70)
**Religion**	*χ*^2^ = 241.64*	*χ*^2^ = 253.17*	*χ*^2^ = 610.78*
Hindu	28.28 (27.93–28.64)	23.36 (23.04–23.68)	4.93 (4.78–5.08)
Muslim	36.30 (35.33–37.29)	27.76 (26.93–28.59)	8.55 (8.06–9.06)
Others	27.47 (26.22–28.75)	23.02 (21.84–24.25)	4.44 (4.05–4.88)
**Mass media exposure**	*χ*^2^ = 4834.84*	*χ*^2^ = 1711.01*	*χ*^2^ = 3808.92*
No	42.02 (41.4–42.63)	31 (30.45–31.56)	11.02 (10.64–11.41)
Yes	24.96 (24.59–25.33)	21.5 (21.16–21.84)	3.46 (3.32–3.59)
**Family Size**	*χ*^2^ = 3509.31*	*χ*^2^ = 1337.04*	*χ*^2^ = 2585.28*
< 5	19.21 (18.67–19.75)	18.05 (17.53–18.58)	1.16 (1.04–1.29)
5–7	32.97 (32.51–33.42)	26.34 (25.93–26.75)	6.63 (6.41–6.86)
> 7	34.59 (33.94–35.24)	26.37 (25.80–26.95)	8.22 (7.87–8.58)
**Age at marriage**	*χ*^2^ = 5258.15*	*χ*^2^ = 3781.22*	*χ*^2^ = 892.21*
< 18	40.86 (40.27–41.45)	33.05 (32.50–33.60)	7.81 (7.52–8.11)
18 or higher	24.06 (23.7–24.42)	19.70 (19.38–20.03)	4.36 (4.20–4.52)
**Type of residence**	*χ*^2^ = 1181.33*	*χ*^2^ = 580.71*	*χ*^2^ = 558.96*
Urban	23.57 (22.83–24.32)	20.13 (19.49–20.78)	3.44 (3.16–3.74)
Rural	31.86 (31.49–32.23)	25.58 (25.25–25.91)	6.28 (6.11–6.46)
**Region of Residence**	*χ*^2^ = 1874.25*	*χ*^2^ = 696.92*	*χ*^2^ = 1582.35*
South	23.26 (22.50–24.03)	21.40 (20.67–22.14)	1.86 (1.65–2.11)
North	27.39 (26.75–28.05)	22.79 (22.19–23.39)	4.61 (4.32–4.91)
Central	34.69 (34.11–35.28)	26.90 (26.39–27.42)	7.79 (7.48–8.11)
East	32.87 (32.15–33.59)	26.06 (25.42–26.71)	6.81 (6.47–7.17)
Northeast	26.28 (25.41–27.17)	20.43 (19.66–21.22)	5.85 (5.46–6.27)
West	23.70 (22.47–24.97)	20.07 (19.03–21.14)	3.63 (3.21–4.11)
**Status of last birth**	*χ*^2^ = 4119.71*	*χ*^2^ = 2192.47*	*χ*^2^ = 1659.40*
Wanted	27.46 (27.13–27.80)	22.64 (22.34–22.94)	4.83 (4.68–4.97)
Unwanted	53.16 (51.99–54.33)	40.20 (39.10–41.32)	12.96 (12.21–13.74)
**Place of delivery**	*χ*^2^ = 4330.35*	*χ*^2^ = 1751.38*	*χ*^2^ = 2841.94*
Health facility	27.14 (26.80–27.49)	22.63 (22.32–22.94)	4.52 (4.38–4.66)
Home	51.02 (50.06–51.98)	36.85 (35.96–37.75)	14.16 (13.51–14.84)
**Type of delivery**	*χ*^2^ = 3358.44*	*χ*^2^ = 1687.46*	*χ*^2^ = 1523.02*
Normal	33.05 (32.67–33.44)	26.39 (26.05–26.73)	6.66 (6.48–6.85)
Caesarean	18.32 (17.78–18.87)	16.61 (16.09–17.13)	1.71 (1.54–1.91)
**ANC visits**	*χ*^2^ = 2912.01*	*χ*^2^ = 1426.96*	*χ*^2^ = 1386.93*
< 4	36.47 (35.96–36.99)	28.60 (28.15–29.07)	7.87 (7.61–8.14)
4 or more	24.58 (24.17–25.00)	20.81 (20.43–21.19)	3.78 (3.62–3.94)
**Currently using contraceptive methods**	*χ*^2^ = 486.63*	*χ*^2^ = 354.65*	*χ*^2^ = 78.51*
Yes	31.50 (31.07–31.94)	25.63 (25.24–26.02)	5.87 (5.68–6.07)
No	26.63 (26.13–27.13)	21.73 (21.29–22.18)	4.90 (4.68–5.12)

*p < 0.001

Women belonging to the poorest wealth index group showed highest prevalence of single (31.6%) and multiple HRFB (11.21%), and the prevalence decreases significantly as we move upward in the wealth status. A similar pattern is noticed for educational level with the highest prevalence of single (35.6%) and multiple HRFB (15.96%) among women with no education and lowest prevalence among women with higher education. The prevalence of single and multiple HRFB is found to be higher in women belonging to SC and ST social groups. Considering religion, Muslim women had higher exposure to single (27.76%) as well as multiple HRFB (8.55%) compared to other women. Higher prevalence of HRFB was observed among women who has no exposure to mass media compared to their counterparts. Women living in larger families had higher share of high-risk births compared to those living in smaller families. Notably, the prevalence of HRFB is found to be higher among women who married before the age of 18 years (56.94%) and among women who had unwanted birth (56.94%). Both single and multiple HRFB prevalence was found to be higher among rural women and women living in central and eastern region of the country.

### Predictors of HRFB prevalence

Table 5 presents the results of the multivariate analysis. Model I is a binomial logistic regression model that presents the odds of any HRFB behavior, while Model II is a multinomial logistic regression model that provides relative risk ratios for single and multiple HRFB among women across all selected socio-economic and demographic variables. In both models, no HRFB was considered as the reference category.

**Table 5:** Socio-economic and demographic predictors of high-risk fertility behaviour among married women in India, NFHS-5 (2019–21)

Background Characteristics	Model I (Binomial Logistic)	Model II (Multinomial Logistic)	
	Any HRFB vs No HRFB OR (95% CI)	Single HRFB vs No HRFB RRR (95% CI)	Multiple HRFB vs No HRFB RRR (95% CI)
**Socio-economic Characteristics**			
**Wealth Index**			
Poorest®	1.00	1.00	1.00
Poorer	0.87 (0.85–0.90)*	0.89 (0.87–0.93)*	0.79 (0.75–0.84)*
Middle	0.80 (0.77–0.83)*	0.84 (0.80–0.87)*	0.66 (0.62–0.72)*
Richer	0.71 (0.69–0.75)*	0.76 (0.73–0.79)*	0.53 (0.48–0.58)*
Richest	0.60 (0.58–0.64)*	0.65 (0.61–0.69)*	0.40 (0.35–0.45)*
**Educational level**			
No education®	1.00	1.00	1.00
Primary	0.68 (0.65–0.70)*	0.75 (0.72–0.78)*	0.49 (0.46–0.52)*
Secondary	0.44 (0.42–0.45)*	0.53 (0.51–0.55)*	0.19 (0.17–0.21)*
Higher	0.29 (0.28–0.31)*	0.38 (0.36–0.40)*	0.08 (0.07–0.09)*
**Social group**			
General®	1.00	1.00	1.00
SC	1.16 (1.12–1.20)*	1.13 (1.10–1.17)*	1.42 (1.32–1.55)*
ST	1.08 (1.04–1.13)*	1.05 (1.01–1.09)*	1.33 (1.23–1.45)*
OBC	1.10 (1.06–1.13)*	1.07 (1.04–1.10)*	1.27 (1.19–1.37)*
**Religion**			
Hindu®	1.00	1.00	1.00
Muslim	1.36 (1.32–1.41)*	1.29 (1.25–1.34)*	1.80 (1.70–1.92)*
Others	1.79 (1.71–1.86)*	1.63 (1.56–1.70)*	2.71 (2.50–2.94)*
**Mass media exposure**			
No®	1.00	1.00	1.00
Yes	0.89 (0.86–0.91)*	0.92 (0.89–0.95)*	0.77 (0.73–0.81)*
**Demographic Characteristics**			
**Family Size**			
< 5®	1.00	1.00	1.00
5–7	2.08 (2.02–2.14)*	1.78 (1.73–1.83)*	6.11 (5.65–6.61)*
> 7	2.43 (2.35–2.51)*	1.94 (1.88–2.01)*	9.67 (8.89–10.52)*
**Age at marriage**			
< 18®	1.00	1.00	1.00
18 or higher	0.65 (0.63–0.67)*	0.62 (0.60–0.63)*	0.84 (0.80–0.88)*
**Type of residence**			
Urban®	1.00	1.00	1.00
Rural	0.90 (0.86–0.92)*	0.92 (0.89–0.95)*	0.79 (0.73–0.84)*
**Region of Residence**			
South®	1.00	1.00	1.00
North	0.79 (0.75–0.82)*	0.78 (0.75–0.82)	1.02 (0.92–1.15)*
Central	0.91 (0.87–0.95)*	0.87 (0.84–0.91)*	1.42 (1.28–1.58)*
East	0.71 (0.69–0.75)*	0.70 (0.67–0.74)	1.01 (0.90–1.13)*
Northeast	0.71 (0.67–0.74)*	0.65 (0.62–0.69)*	1.31 (1.16–1.48)*
West	0.78 (0.74–0.83)*	0.77 (0.73–0.81)	1.10 (0.96–1.25)*
**Reproductive characteristics**			
**Status of last birth**			
Wanted®	1.00	1.00	1.00
Unwanted	2.61 (2.52–2.72)*	2.47 (2.37–2.57)*	3.37 (3.16–3.59)*
**Place of Delivery**			
Health facility®	1.00	1.00	1.00
Home	1.56 (1.51–1.61)*	1.49 (1.44–1.54)*	1.75 (1.66–1.84)*
**Type of Delivery**			
Normal®	1.00	1.00	1.00
Caesarean	0.77 (0.75–0.79)*	0.80 (0.77–0.82)*	0.57 (0.53–0.62)*
**ANC Visits**			
< 4®	1.00	1.00	1.00
4 or more	0.83 (0.81–0.85)*	0.83 (0.81–0.85)*	0.80 (0.77–0.84)*
**Currently using contraceptive methods**			
No®	1.00	1.00	1.00
Yes	0.74 (0.72–0.76)*	0.75 (0.73–0.77)*	0.71 (0.68–0.74)*

*OR* Odds Ratios, *RRR* Relative Risk Ratio, *CI* Confidence Interval

*p < 0.001

Model I reveals that the likelihood of high-risk births significantly decreases with an increase in household wealth status and women’s education level. Richest women are 40% less likely to experience any HRFB compared to the poorest women (OR = 0.60; 95% CI: 0.58–0.64), and higher-educated women have 71% less likelihood of HRFB compared to illiterate women (OR = 0.29; 95% CI: 0.28–0.31). Women belonging to the SC category showed the highest odds of HRFB, around 1.2 times more than general-category women (OR = 1.16; 95% CI: 1.12–1.20). Muslim women were 1.4 times more likely to have high-risk births (OR = 1.36; 95%CI: 1.32–1.41), and women belonging to other religions showed 1.8 times more likelihood of HRFB compared to Hindu women (OR = 1.79; 95% CI: 1.71–1.86). Exposure to mass media decreases the likelihood of high-risk births by 11% (OR = 0.89; 95% CI: 0.86–0.91). The likelihood of HRFB rose by 2.1 times (OR = 2.08; 95% CI: 2.02–2.14) and 2.4 times (OR = 2.43; 95% CI: 2.35–2.51) for women residing in households with family sizes of 5–7 and more than 7, respectively, compared to those living in smaller families. The likelihood of any HRFB was reduced by a factor of 0.65 among women who got married after the age of 18 years (OR = 0.65; 95% CI: 0.63–0.67). Regarding type and region of residence, rural women showed a lower likelihood of HRFB than those living in urban areas (OR = 0.90; 95% CI: 0.86–0.92), and women residing in the eastern (OR = 0.71; 95% CI: 0.69–0.75) and northeastern regions (OR = 0.71; 95% CI: 0.67–0.74) exhibited the lowest likelihood of HRFB, which is 29% less compared to those residing in the southern region. The likelihood of high-risk births was increased by a factor of 2.6 among women who had unwanted births (OR = 2.61; 95% CI: 2.52–2.72). Women who delivered at home showed 1.6 times more likelihood of HRFB (OR = 1.56; 95% CI: 1.51–1.61). Finally, the likelihood of any HRFB was significantly lower among women who had a caesarean delivery (OR = 0.77; 95% CI: 0.75–0.79), received four or more ANC consultations (OR = 0.83; 95% CI: 0.81–0.85), and had been using contraceptive methods (OR = 0.74; 95% CI: 0.72–0.76).

Similar to Model I, Model II exhibited a significant rise in the relative risk of both single and multiple HRFB as we move upward in women’s wealth status and educational level. The relative risk of single HRFB was reduced by a factor of 0.65 among the richest women (RRR = 0.65; 95% CI:0.61–0.69), and the relative risk of multiple HRFB was observed to be reduced by a factor of 0.4 among the wealthiest (RRR = 0.40; 95% CI:0.35–0.45), compared to the poorest women. Concerning educational levels, the relative risk of single HRFB decreased by 62% among women with higher educational levels (RRR = 0.38; 95% CI:0.36–0.40), while the relative risk of multiple HRFB decreased by 92% among higher-educated women (RRR = 0.08; 95% CI:0.07–0.09), as compared to illiterate women. Belonging to a social group other than the general category and being non-Hindu are significantly associated with a higher likelihood of both single and multiple HRFB among women. For instance, the relative risk of single HRFB was 13% higher (RRR = 1.13; 95% CI:1.10–1.17), and for multiple HRFB, it was 42% higher (RRR = 1.42; 95% CI:1.32–1.55) among SC women compared to women in the general category. Likewise, the relative risk of experiencing single HRFB was 1.3 times higher (RRR = 1.29; 95% CI:1.25–1.34), and for multiple HRFB, it was 1.8 times higher (RRR = 1.80; 95% CI:1.70–1.92) among Muslim women compared to Hindu women. The relative risk of experiencing single and multiple HRFB decreased by 8% (RRR = 0.92; 95% CI:0.89–0.95) and 23% (RRR = 0.77; 95% CI:0.73–0.81), respectively, among women who were exposed to mass media compared to those who were not. Women residing in smaller families, with fewer than 5 household members, exhibited a reduced likelihood of both single and multiple HRFB in comparison to their counterparts. The relative risk of single HRFB increased by 78% for women living in households with a family size of 5–7 (RRR = 1.78; 95% CI:1.73–1.83) and by 94% for those living in households with a family size greater than 7 (RRR = 1.94; 95% CI:1.88–2.01). For multiple HRFB, the relative risk is approximately sixfold (RRR = 6.11; 95% CI:5.65–6.61) and tenfold (RRR = 9.67; 95% CI: 8.89–10.52) higher among women living in households with a family size of 5–7 and greater than 7, respectively, in comparison to those living in smaller families. Marrying after the age of 18 years was associated with a lower relative risk of both single (RRR = 0.62; 95% CI:0.60–0.63) and multiple HRFB (RRR = 0.84; 95% CI:0.80–0.88) compared to their corresponding counterparts. Unlike all the other background characteristics, the region of residence exhibited a contrasting association for single and multiple HRFB. For instance, in the central region, the relative risk of single HRFB was observed to be reduced by a factor of 0.87 (RRR = 0.87; 95% CI:0.84–0.91), whereas for multiple HRFB, it increased by a factor of 1.42 (RRR = 1.42; 95% CI:1.28–1.58), compared to women living in the southern region. Similarly, for women residing in the northeastern region, the relative risk of single HRFB was 35% lower (RRR = 0.65; 95% CI:0.62–0.69), while it was 31% higher for multiple HRFB (RRR = 1.31; 95% CI:1.16–1.48), compared to those living in the southern region of the country. In terms of the reproductive characteristics of the women, the relative risk of single HRFB was approximately 2.5 times higher (RRR = 2.47; 95% CI:2.37–2.57) and for multiple HRFB, it was more than 3 times higher (RRR = 3.37: 95% CI:3.16–3.59) among those who had an unwanted birth. The women who delivered at home had 1.5 times greater relative risk of single HRFB (RRR = 1.49; 95% CI:1.44–1.54) and 1.8 times higher relative risk for multiple HRFB (RRR = 1.75; 95% CI:1.66–1.84) compared to those who delivered at a health facility. The relative risk of single HRFB significantly decreased for women who underwent caesarean delivery (RRR = 0.80; 95% CI:0.77–0.82), received four or more ANC consultations (RRR = 0.83; 95% CI:0.81–0.85), and utilized contraceptive methods (RRR = 0.75; 95% CI:0.73–0.77). Likewise, the relative risk of experiencing multiple HRFB was 43% lower for women who had a caesarean delivery (RRR = 0.57; 95% CI:0.53–0.62), 20% lower for those who had four or more ANC visits (RRR = 0.80; 95% CI:0.77–0.84), and 29% lower for those who were using contraceptive methods (RRR = 0.71; 95% CI:0.68–0.74).

## Discussion

The findings of the study reveal that the prevalence of HRFB has shown an overall declining trend from 1992 to 93 to 2019–21 in India. However, 29.56% of married women are still experiencing high-risk births, with significantly higher rates in several states. Meghalaya ranked as the most disadvantaged state concerning the proportion of women with High-Risk Fertility Behavior (HRFB) at 49.85%, followed by Bihar at 46.41% and Mizoram at 40.54. In contrast, Odisha demonstrated the lowest prevalence of HRFB at 16.8%, with Sikkim closely following at 17.1%. The inter-state variation observed in this study can be attributed to differentials in socio-demographic, economic, and health indicators, as well as health infrastructure among states. The central and eastern region includes states like Bihar, Jharkhand, Madhya Pradesh, Chhattisgarh, and Uttar Pradesh which are recognized as the Empowered Action Group (EAG) states by the Government of India, as they significantly lag behind the more prosperous southern and western states in many human development and health indicators. Similarly, India’s northeastern region consists of poorly developed and tribal-dominated states.

The findings of bivariate and multivariate analysis indicated that socio-economic factors, such as wealth index, educational level, social group, religion, mass media exposure, demographic factors such as age at marriage, family size, type and region of residence, and reproductive factors such as whether wanted the last birth, place of delivery, type of delivery, number of ANC visits and current use of contraceptive methods, are significant predictors of high-risk births in India.

Women’s economic and educational status appears to have a strong impact on the HRFB prevalence. In line with previous studies, the present study revealed that belonging to economically sound households reduces the odds of maternal HRFB [30–34]. Wealth status is inversely related to early childbearing, with studies consistently showing that women from lower socio-economic backgrounds are more likely to have their first child at a young age [40, 41]. This relationship can be attributed to limited educational opportunities, reduced access to healthcare, and lower awareness of contraception methods. Previous studies have also indicated that women with lower wealth status tend to have high parity and a shorter interpregnancy interval. This is partly attributed to limited access to family planning services and, in some cases, to higher son preference among the poor. Women in economically disadvantaged households often have less control over their reproductive decisions, leading to a pattern of rapid repeat pregnancies and larger family sizes [42–45].

Concerning educational level, a sharp decrease in the odds of HRFB was observed as we moved from women with no education to highly educated women. Numerous prior studies have also emphasized the pivotal role of education in this context [30–34]. A higher educational level is typically correlated with older age at marriage and a lower number of children. Educated women possess greater knowledge about birth spacing and limitation, along with increased awareness of pregnancy and childbirth-related risk factors [46–48]. Thus, improving women’s educational levels has the potential to address the issue of high-risk births. Additionally, educated mothers are more empowered to make informed decisions about the well-being of themselves and their children [49].

Regarding socioreligious groups, Muslim, SC, and ST women were more likely to have high-risk births. The reluctance of Muslim women to use contraceptive methods, influenced by religious beliefs, may contribute to the higher prevalence of HRFB among them. Furthermore, a substantial unmet need for family planning is evident among Muslim women. Moreover, Muslims are generally socioeconomically underprivileged in India, with a lower literacy rate and a higher proportion of the population living under the poverty line [39, 50–53]. Similarly, SC and ST are among the most socially marginalized and socio-economically disadvantaged social groups in India. Thus, there could be many possible explanations for higher HRFB among women from such deprived and backward classes, for instance, lack of awareness about maternal and child health care, lack of access to health information and health care resources, and less benefit from existing public health policies [54, 55].

Consistent with some previous similar studies, this study also highlighted the importance of media exposure in the reduction of high-risk births with the finding that women unexposed to mass media had significantly higher odds of HRFB than those who were exposed to media [30, 31, 33]. Another important finding of this study was the influence of age at marriage on maternal HRFB. Being married after the age of 18 years was found to be a protective factor for high-risk births. Existing literature suggests that early marriage in developing countries often results in a low level of education and low autonomy, which could be a plausible reason for the higher prevalence of HRFB among women who got married before 18 years [56, 57].

Another noteworthy observation derived from this study is that the reproductive characteristics of women significantly influence high-risk births among them. For instance, women who had unwanted births demonstrated a higher likelihood of engaging in high-risk fertility behavior compared to those with previously desired pregnancies. This emphasizes the role of family planning in the prevention of high-risk births. The study also revealed that high-risk births were more likely to occur among women who were not using any contraceptive methods. This result may be attributed to unwanted births resulting from the non-utilization of contraceptive methods. In terms of place of delivery, women delivering at home exhibited a higher prevalence of high-risk fertility behaviors compared to those delivering at a health facility. Regarding the type of delivery, women who underwent caesarean delivery were less likely to experience HRFB compared to those who had normal delivery during their last childbirth. This can be ascribed to the observation that women delivering via caesarean section were less inclined to have additional children compared to those delivering vaginally. Besides, studies have reported caesarean section deliveries to be associated with a higher probability of actively pursuing contraception following childbirth, potentially leading to lower odds of HRFB [58]. Having four or more ANC consultations emerged as a protective factor against HRFB among women. This result can be attributed to the fact that antenatal care offers opportunities to provide pregnant women with a range of interventions crucial to their health and well-being during childbearing. Consequently, they are more likely to receive information regarding the significance of routine check-ups, maternal nutrition, delivery complications, and the consequences of engaging in high-risk fertility behavior [59, 60]. These findings on the reproductive characteristics of women align with previous comparable studies [30–33].

This study emphasizes the urgent need for the implementation of comprehensive programmatic interventions aimed at preventing high-risk births. It is imperative to direct health interventions toward states with a high prevalence of High-Risk Fertility Behavior (HRFB) and the socioeconomically disadvantaged segments of the population. Proactive measures should be taken to enhance awareness regarding the HRFB among women, particularly those who are economically disadvantaged, have limited education, or belong to socially marginalized groups. The role of mass media in disseminating this information should not be underestimated, as it can significantly contribute to raising awareness.

In addition to targeted intervention programs, integrating information on HRFB and its potential consequences into existing family planning initiatives and maternal and child health programs, including child immunization visits, is crucial. This integration would ensure that women and their family members receive comprehensive knowledge about the risks involved and the importance of making informed reproductive choices. By leveraging existing platforms, such as family planning services and routine health check-ups, a broader population can be reached to educate and empower women. This personalized approach not only addresses the immediate concerns but also contributes to a long-term reduction in the incidence of HRFB. In summary, a multifaceted strategy involving targeted interventions, mass media engagement, integration into existing healthcare programs, and personalized family planning services is vital for mitigating the prevalence of high-risk births and promoting reproductive health in vulnerable populations.

Considering the strengths of this study, to the best of our knowledge, this is the first study to demonstrate the trends and patterns in HRFB prevalence and its associated predictors in India. Another major strength was the utilization of the most recent nationally representative survey data. Therefore, the findings of this study can be generalized to all women of reproductive age (15–49 years) in India, providing policymakers with better evidence for implementing appropriate interventions.

However, the study also has some limitations. The NFHS is susceptible to recall and social desirability bias and hence the precision of the estimates depends on the quality of reporting. Since the dataset is cross-sectional in nature, it is difficult to access causality. Moreover, information recorded at the time of the interview, such as socioeconomic confounders, may fail to accurately reflect the true conditions at the time of childbearing.

## Conclusion

The study has highlighted the persistently high prevalence of high-risk fertility behavior among Indian women, revealing socioeconomic and demographic determinants. Despite a gradual national decline over three decades, certain states and population segments still exhibit notably high rates. Protective factors such as higher maternal education, affluent family backgrounds, smaller family sizes, marriage after the age of 18, and desired births are found to be associated with lower instances of HRFB. The findings underscore the need for targeted interventions, emphasizing a necessary overhaul of existing programs.

Tailoring efforts to the socioeconomically disadvantaged is crucial, focusing on education and awareness about reproductive health and associated risks. Addressing the specific needs of vulnerable populations is key to ensuring an equitable distribution of reproductive health knowledge. States with higher rates of high-risk births should prioritize health infrastructure improvements, strengthening healthcare systems to provide better access to maternal and reproductive healthcare services. This comprehensive approach is essential for promoting reproductive health and minimizing adverse pregnancy outcomes.

## Data Availability

The dataset analysed during the current study are available upon request from the Demographic and Health Surveys (DHS) repository, https://dhsprogram.com/data/available-datasets.cfm

## Abbreviations

CEB: Census Enumeration Block
DHS: Demographic Health Survey
HRFB: High-Risk Fertility Behaviour
IR: Individual Record
NFHS: National Family Health Survey OBC Other Backward Class
PSU: Primary Sampling Unit
SC: Scheduled Class
ST: Scheduled Tribe
UT: Union Territory
